# Conflict-sensitive neurons gate interocular suppression in human visual cortex

**DOI:** 10.1038/s41598-018-19809-w

**Published:** 2018-01-19

**Authors:** Sucharit Katyal, Mark Vergeer, Sheng He, Bin He, Stephen A. Engel

**Affiliations:** 1 Department of Psychology, University of Minnesota Twin Cities, Minneapolis, MN 55455 USA; 2 Department of Biomedical Engineering, University of Minnesota Twin Cities, Minneapolis, MN 55455 USA

## Abstract

Neural suppression plays an important role in cortical function, including sensory, memory, and motor systems. It remains, however, relatively poorly understood. A paradigmatic case arises when conflicting images are presented to the two eyes. These images can compete for awareness, and one is usually strongly suppressed. The mechanisms that resolve such interocular conflict remain unclear. Suppression could arise solely from “winner-take-all” competition between neurons responsive to each eye. Alternatively, suppression could also depend upon neurons detecting interocular conflict. Here, we provide physiological evidence in human visual cortex for the latter: suppression depends upon conflict-sensitive neurons. We recorded steady-state visual evoked potentials (SSVEP), and used the logic of selective adaptation. The amplitude of SSVEP responses at intermodulation frequencies strengthened as interocular conflict in the stimulus increased, suggesting the presence of neurons responsive to conflict. Critically, adaptation to conflict both reduced this SSVEP effect, and increased the amount of conflict needed to produce perceptual suppression. The simplest account of these results is that interocular-conflict-sensitive neurons exist in human cortex: adaptation likely reduced the responsiveness of these neurons which in turn raised the amount of conflict required to produce perceptual suppression. Similar mechanisms may be used to resolve other varieties of perceptual conflict.

## Introduction

Neural signals in the brain often conflict. A memory cue might prompt recollection of two memories, only one of which applies to the current situation; a graspable object might activate multiple motor plans, only one of which is executed. To produce consistent behavior, the brain may suppress one of the two signals.

In perception, binocularity can present a similar challenge when the eyes receive incompatible information. Conflict between responses originating in the two eyes occurs commonly in natural viewing, for example when attending peripheral objects that are occluded, in one eye only, by the nose. Binocular conflict can make it difficult to interpret the content of a scene, and to perform actions dependent upon a single interpretation. To cope with this problem, the visual system suppresses information originating from one of the two eyes, when the images they receive are sufficiently different. In some circumstances, the suppression alternates between the two eyes’ inputs. This phenomenon, termed binocular rivalry, has a long history of laboratory study^1–3^, serving as a model system for neural suppression.

The neural processes underlying interocular suppression remain actively debated. Signals from the two retinae are largely segregated in the lateral geniculate nucleus, and in the input layers of primary visual cortex (V1), where neurons respond preferentially to stimulation of one eye. Inhibition of these monocular neurons in V1 is likely responsible for some of the perceptual suppression that occurs during rivalry^4–6^, though inhibition of neurons in later stages of processing may also play a role^6,7^.

Two theories can account for suppression of monocular neuronal responses in V1. One class of models suggests that rivalry involves mutual inhibition between neurons that are selective for different combinations of features and eyes. For example, neurons whose preferred input is horizontal features in the left eye could suppress neurons that prefer vertical features in the right eye. Many models of rivalry contain this “winner-take-all” mechanism for suppression^4,8–11^.

However, these models sometimes incorrectly predict strong suppression, in part because suppression is passive, and occurs automatically, regardless of whether the images in the two eyes match or are in conflict. For example, when identical plaid patterns consisting of horizontal and vertical orientations are presented to both eyes, monocular neurons preferring vertical stimulation to the left eye will inhibit neurons that prefer horizontal stimulation to the right eye, even though interocular conflict is absent in the stimulus.

A recent alternative view proposes that active mechanisms measure conflict between the two eyes, and enable interocular suppression if the conflict is large^12^. Said and Heeger^12^ showed how neurons that serve as interocular conflict detectors could reduce inappropriate suppression in models of binocular rivalry.

Physiological evidence for interocular conflict detecting neurons in humans remains sparse, however (see Discussion). As a first test for the presence of such neurons, Katyal *et al*.^13^ measured the steady-state evoked potential (SSVEP) with and without interocular conflict present in the visual stimulus. To produce the SSVEP signals, they presented test stimuli to each eye that flickered at different rates, a “frequency-tagging” approach that was successfully used in prior work on binocular rivalry^14–17^. Notably, responses at intermodulation frequencies, which are sums and differences of integer multiples of the fundamental frequencies presented to each eye, could only arise from neurons that (nonlinearly) combine input from the two eyes^18,19^. Energy at these frequencies increased when interocular conflict was introduced to the stimulus, while energy at the fundamentals did not. The increase in intermodulation response could have arisen from conflict-sensitive neurons increasing their activity in the presence of interocular stimulus conflict.

Here, we build upon these results to provide the strongest neurophysiological evidence to date for the existence of conflict-sensitive neurons that regulate suppression in human cortex. We first replicated the increase in intermodulation response with interocular conflict. We then tested whether the increase arose from neurons sensitive to conflict by using the logic of selective adaptation: If conflict-sensitive neurons exist, then prolonged exposure to interocular conflict should reduce their responsiveness, which should lower the signal in the SSVEP attributable to them.

Our results confirmed this prediction: the increase in intermodulation response with stimulus conflict was substantially reduced following adaptation to conflicting stimuli. Additionally, if these neurons are important for perception, then their reduced responsiveness should make rivalry harder to initiate. This prediction was confirmed in a parallel behavioral experiment; following adaptation to conflict, a greater amount of it was required before perceptual suppression occurred.

## Results

**Figure 1:**
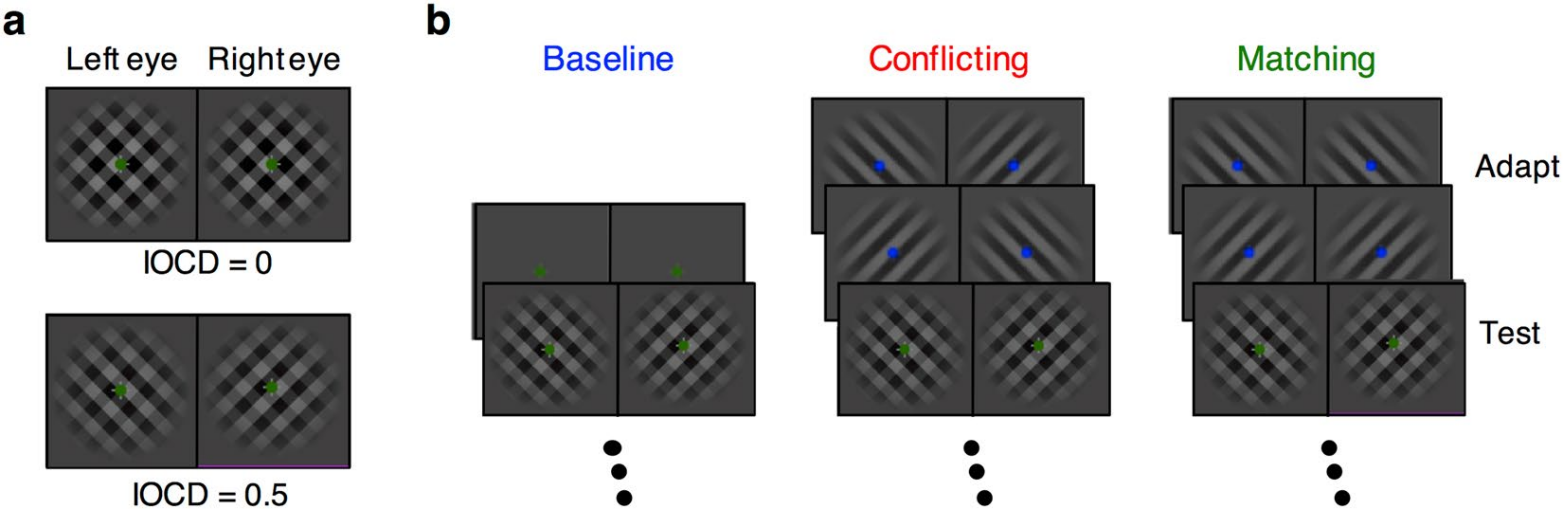
Stimuli used in the experiments. (**a**) Two sample levels of interocular contrast difference (IOCD): 0 (matching plaids), and 0.5 (conflicting plaids). (**b**) Baseline and the two adaptation conditions from left to right: baseline (no adaptation), conflicting (rivalry), and matching (fusion). Baseline runs had test stimuli interspersed with presentation of a mean gray field. Conflicting and matching adaptation runs had a 60s initial adaptation period followed by trials containing “top-up” adaptation followed by a test presentation. Adaptors alternated between orthogonal orientations every 1s.

The experimental paradigm is illustrated in Fig. 1. We recorded responses to plaid patterns (Fig. 1a). Interocular conflict was produced in these patterns by introducing a difference between the contrasts of the component gratings in each eye. We term this difference the interocular contrast difference, or IOCD. Figure 1a, for example, shows two such stimuli. For IOCD = 0 (upper panel), the two eyes receive the identical plaid, and there is no conflict. For IOCD = 0.5 (lower panel), the +45° and −45° gratings in the left and right eyes respectively are reduced in contrast by 50%. The +45° grating is now higher contrast in the left eye, while the −45° grating is now higher contrast in the left eye, which produces interocular conflict.

We measured steady state visually evoked EEG responses to test patterns with IOCD = 0 (identical stimuli) and 0.2 (20% difference in the contrast of components in each eye). To evoke “frequency-tagged” SSVEP signals, throughout the experiment, the visual stimulus alternated with a mean gray field at 7.2 Hz (*f*_1_) in the left eye, and 12 Hz (*f*_2_) in the right eye

Responses were measured in three conditions (Fig. 1a). In a baseline condition, 2-s test patterns were interspersed with presentations of a gray field every 950–1150 ms (left panel). In two other adaptation conditions, responses to the test patterns were measured following prolonged exposure to component gratings presented to each eye. These adapters were either conflicting, with orthogonal gratings presented to each eye (Fig. 1a, middle panel), or matching, with identical gratings presented (right panel).

### Behavioral Responses

During data acquisition, subjects performed a discrimination task in order to ensure they maintained attention on the test plaids, which appeared fused at these levels of IOCD (see Discussion). Subjects responded by pressing one of two buttons to indicate whether they saw a higher (IOCD = 0) or lower total contrast plaid (IOCD = 0.2; see Methods). All but one subject performed the task well above chance (mean and SD proportion correct: 90 ± 1%) indicating they were attending the stimuli.

### Basic SSVEP Responses

**Figure 2:**
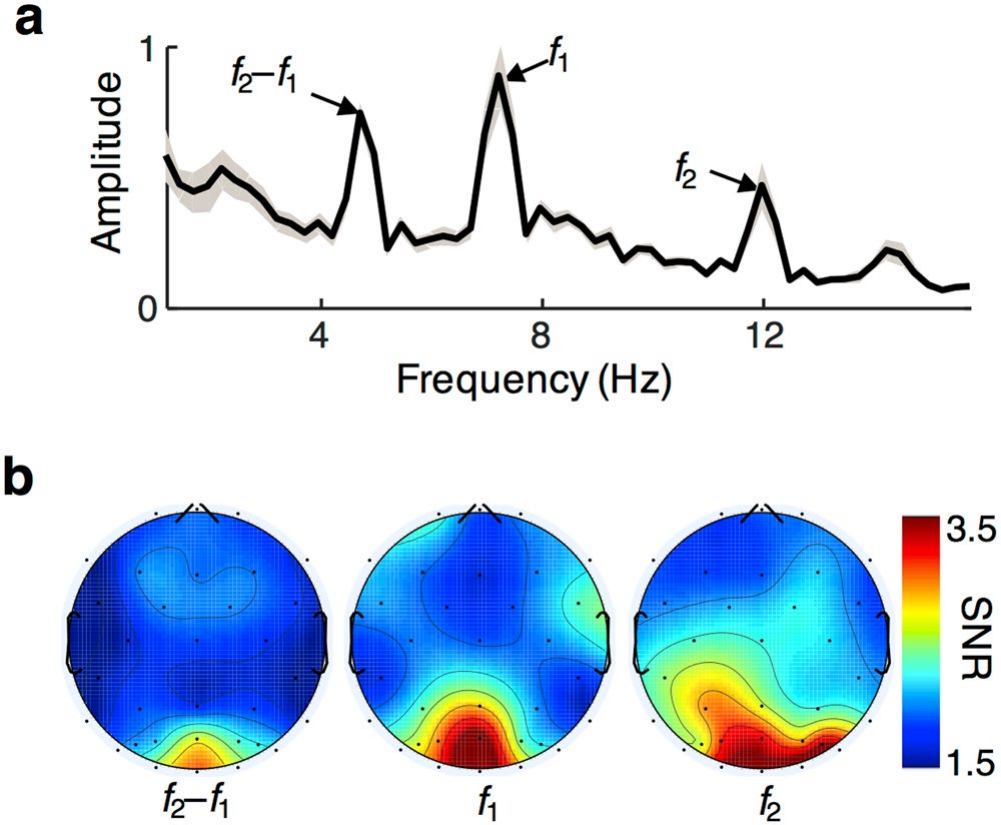
Evoked fundamental and intermodulation frequencies and their topography. (**a**) FFT amplitude (on linear scale) as a function of frequency for the 2-sec test stimuli averaged across conditions, subjects (*N* = 16), and occipital and parieto-occipital electrodes. Gray shaded area indicates standard error of the mean. Fundamental (*f*_1_ and *f*_2_) and intermodulation (*f*_2_–*f*_1_) frequencies indicated. (**b**) Scalp topographies of signal-to-noise ratios at the three frequencies shown in a) averaged across subjects and all three conditions. Maximum amplitudes were evoked at the occipital pole for all three frequencies.

Figure 2a shows the amplitude of the frequency spectrum during the 2-s test stimuli, averaged across all subjects, conditions, and all occipital and parieto-occipital electrodes (see Methods). Strong SSVEP signals were observed at the tagged frequencies (*f*_1_ and *f*_2_), which we term the fundamental frequencies. Strong signals were also present at the difference of these two frequencies, which is one of the intermodulation frequencies. Both kinds of SSVEP responses showed topography consistent with an expected source in early visual cortex (Fig. 2b)^17,20^. Despite differences in raw amplitudes between responses at *f*_1_ and *f*_2_ (7.2 and 12 Hz), their average signal-to-noise ratios (SNR) were comparable.

### Adaptation of conflict-sensitive signals in the SSVEP

**Figure 3:**
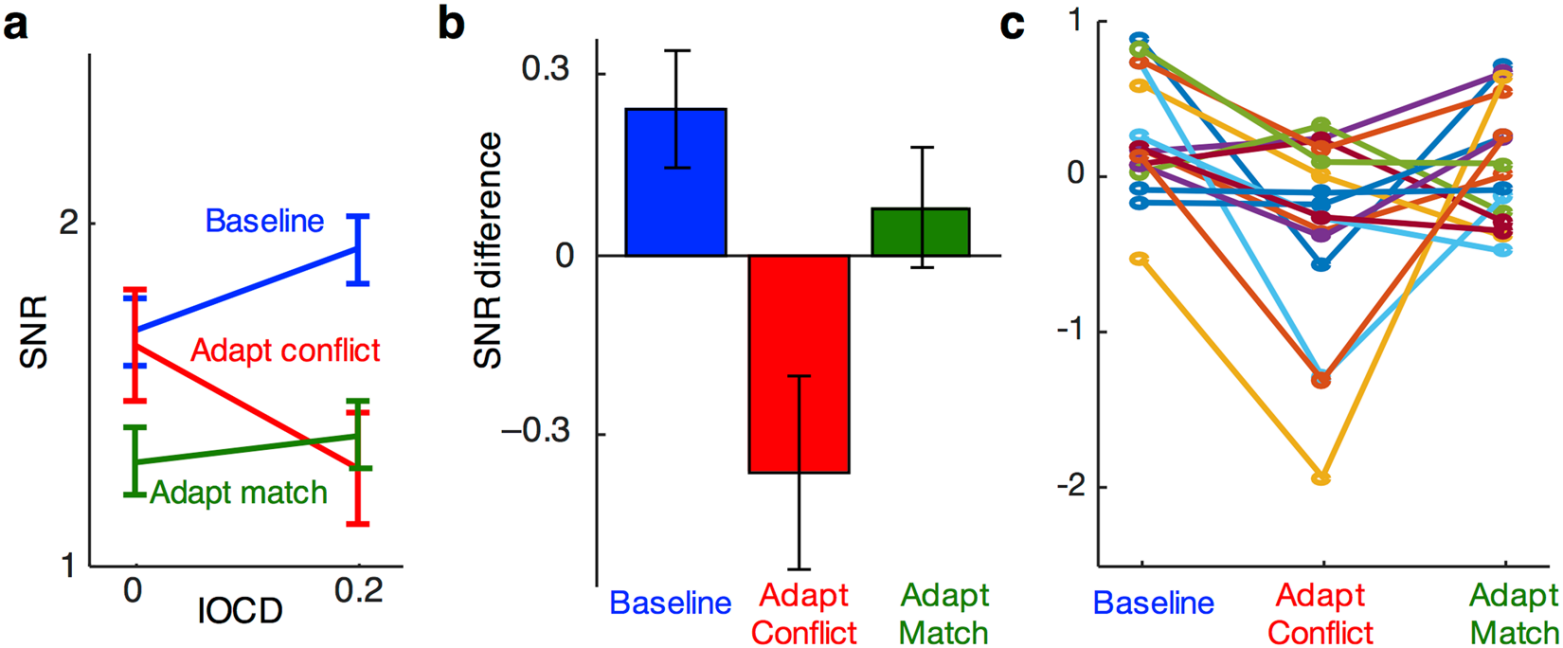
Response at the intermodulation frequency. (**a**) SNR at the SSVEP intermodulation beat frequency (*f*_2_–*f*_1_) for the two levels of test IOCD and three adaptation conditions, averaged across occipital and parieto-occipital electrodes. Error bars are standard errors of the means (*N* = 16) calculated after subtracting the global mean of each subject’s data (across the two IOCDs). (**b**) Difference scores computed by subtracting SNRs of the two test IOCDs for the three adaptation conditions in a). (**c**) Difference scores, as in (**b**), for the sixteen individual subjects.

Our primary analyses focused on intermodulation frequencies, since these can only arise from neurons that integrate information from the two eyes. In the baseline condition, response at the intermodulation frequency *f*_2_–*f*_1_ increased in amplitude as interocular conflict was introduced to the stimulus (Fig. 3a blue). Specifically, as IOCD rose from 0 to 0.2, the intermodulation SNR increased significantly (*t*(15) = 2.5, *p* < 0.05, Cohen’s *d* = 0.61).

This increase in response with stimulus conflict likely arises from conflict-sensitive neurons^13^. Such neurons would be unresponsive when IOCD = 0, due to the lack of stimulus conflict, but would increase their response for IOCD = 0.2, when some interocular conflict is present. Note that reliable intermodulation response was also present when the stimulus contained no interocular conflict (IOCD of 0). This response likely arises from binocular neurons that integrate matching information from the two eyes (See Discussion).

We next tested more directly whether conflict-sensitive neurons were the source of the observed intermodulation response increase. To do so, the experiment used the logic of selective adaptation. We hypothesized that following exposure to rivaling gratings, which causes strong interocular conflict, responses from conflict-sensitive neurons should be reduced. This predicts that the increase in intermodulation signal, measured when IOCD rises from 0 to 0.2 in the baseline condition, should be absent or reduced following adaptation to rivalry. To rule out the possibility that prolonged exposure to *any* stimulus would eliminate the SSVEP response to interocular conflict, we also measured signals following exposure to matching images in the two eyes. Since matching stimuli will not produce large responses in neurons sensitive to conflict, this condition should show a different pattern of responses than adaptation to conflict.

Adaptation to interocular conflict indeed eliminated the increase in intermodulation response as a function of test stimulus conflict (Fig. 3a). To formally compare effects of adaptation in the three conditions, we performed a 2 × 3 ANOVA with factors IOCD (0 and 0.2) and adaptation condition (Baseline, Adapt Conflict, Adapt Match). We found a statistically reliable interaction between the two factors (*F*(15, 2) = 6.3, *p* < 0.01, partial *η*^2^ = 0.30), indicating that adaptation reliably changed the intermodulation response as a function of IOCD. Post-hoc tests showed that adaptation to conflict lowered the effect of IOCD on response, compared to the baseline condition. For these analyses, we computed difference scores by subtracting responses to test stimuli with no conflict (IOCD = 0) from responses to test stimuli containing conflict (IOCD = 0.2; Fig. 3b; note that in a within-subjects design, using difference scores is equivalent to testing for an interaction between the pair of conditions and IOCD). Differences scores were smaller following adaptation to conflict than in baseline (*t*(15) = 3.6, *p* < 0.005, Cohen’s *d* = –0.90; Fig. 3b). This pattern matches what would be expected if adaptation reduced the responsiveness of conflict-sensitive neurons, so that they then failed to respond robustly when conflict was present in the stimulus (i.e. at IOCD = 0.2).

Post-hoc tests also showed a strong trend for adaptation to matching stimuli to produce different effects on response as a function of IOCD than adaptation to conflict (*t*(15) = 2.1, *p* = 0.055, Cohen’s *d* = –0.52). Also unlike adaptation to conflict, adaptation to matching stimuli did not produce effects as a function of IOCD that differed baseline (*t*(15) = 1.2, *p* = 0.263).

The initial ANOVA also showed a main effect of adaptation condition (*F*(15, 2) = 3.81, *p* < 0.05, partial *η*^2^ = 0.20). This was driven by lower overall response following adaptation to matching stimuli, which would be expected if part of the intermodulation response to both levels of IOCD were due to binocular integration neurons (see Discussion). Responses in the adaptation to conflict condition, in contrast, were only lower than baseline levels for IOCD = 0.2. This is likely because conflict-sensitive neurons were relatively unresponsive at IOCD = 0.0, and so their response had little room to drop from baseline levels following adaptation. Effects of adaptation were lower at IOCD = 0.2, where conflict sensitive neurons should be active, compared to IOCD = 0 (*t*(15) = −2.2, *p* < 0.05, Cohen’s *d* = −0.56).

**Figure 4:**
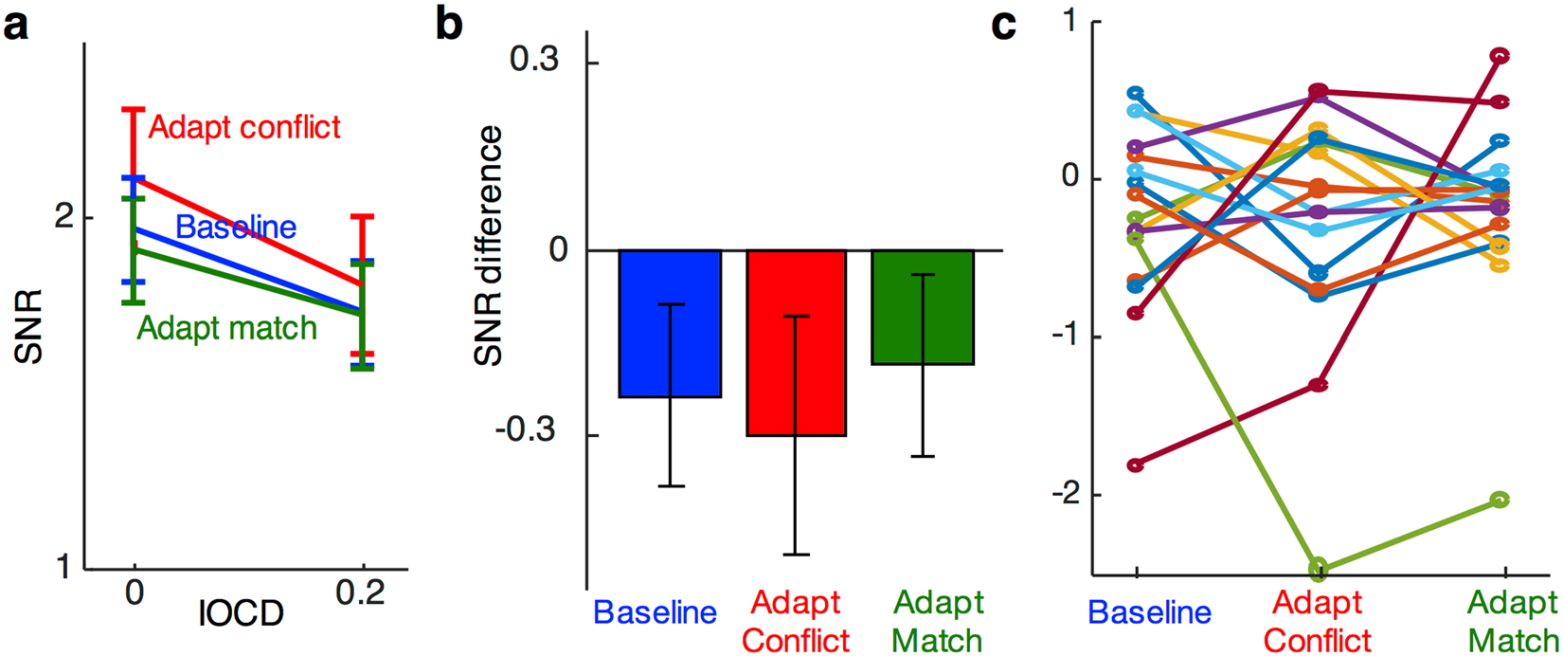
Response at the fundamental frequencies. (**a**) SNR averaged across the two SSVEP fundamental frequencies (*f*_1_ & *f*_2_) for the two levels of test IOCD and three adaptation conditions averaged across occipital and parieto-occipital electrodes. Error bars are standard errors of the means (*N* = 16) calculated after subtracting the global mean from each subject’s data (across the two IOCDs). (**b**) Difference scores between the SNRs for the three adaptation conditions in (**a**). (**c**) Difference scores, as in (**b**), for the sixteen individual subjects.

The selective effect of adaptation to conflict was also specific to the response at intermodulation frequencies, which, as discussed above, can only arise from activation of binocular neurons. SSVEP response at the fundamental frequencies following adaptation (Fig. 4) showed a different pattern than at the intermodulation frequencies (Fig. 3). This observation was confirmed by a 2 × 2 repeated-measures ANOVA on the difference scores, which showed a significant interaction between adaptation type (conflict or matching) and SSVEP frequency (fundamental or intermodulation; *F*(15, 1) = 6.3, *p* < 0.05, partial *η*^2^ = 0.29). Response at fundamental frequencies showed trends towards lower amplitudes as IOCD increased in all three conditions, possibly because the total contrast of the stimulus decreased (see Discussion). A one-way ANOVA of the difference scores showed that the size of this response decrease did not differ between adaptation conditions (*F*(15, 2) = 0.2, *p* = 0.86).

### Behavioral evidence for conflict-sensitive neurons

The EEG results match the predictions of models that contain conflict-sensitive neurons. To test whether such neurons are relevant for perception, we measured the amount of interocular conflict required to initiate perceptual suppression, under the same three adaptation conditions as used for the SSVEP recordings. If signals from conflict-sensitive neurons help initiate interocular suppression, then when adaptation renders them less responsive, greater amounts of conflict should be tolerated before one eye’s pattern is suppressed.

**Figure 5:**
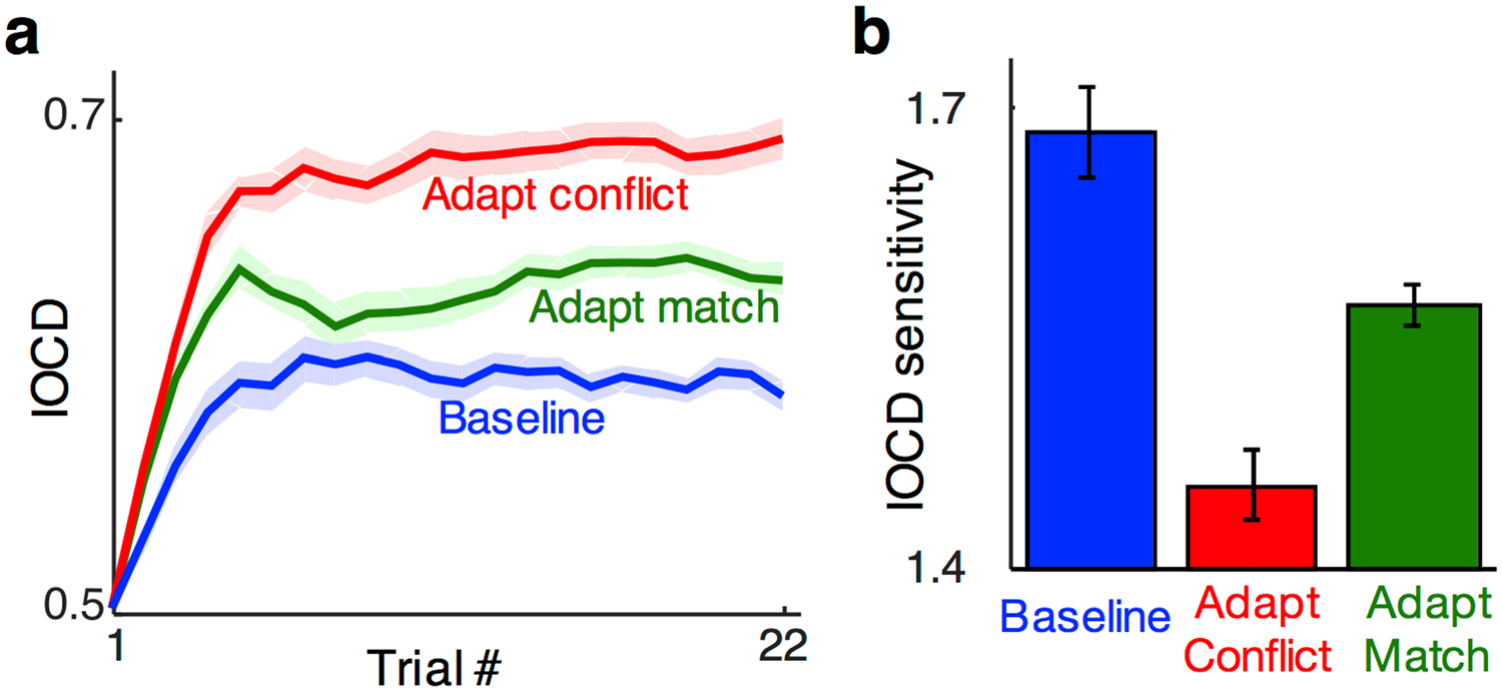
Adaptation thresholds for the behavioural experiment. (**a**) IOCD value presented in behavioural staircases averaged across subjects (*N* = 10) for the three adaptation conditions. Error bars are standard errors of the means calculated after subtracting the global mean of each subject’s data (across the three conditions). (**b**) IOCD sensitivities, 1/threshold, calculated by averaging the second half of the trials in (**a**).

A staircase procedure estimated the IOCD that produced perceptual suppression on 50% of trials, a level that we defined as the suppression threshold. Figure 5a plots average IOCD values for ten subjects during the staircase procedure. The asymptotes of each curve estimate the rivalry threshold IOCD.

Adaptation to conflicting stimuli (red) increased thresholds compared to both baseline (blue) and adaptation to matching stimuli (green). Suppression thresholds were significantly higher following adaptation to rivaling patterns than they were following adaptation to fusible ones (average of last 11 trials; *t*(9) = 5.0, *p* < 0.001, Cohen’s *d* = 1.88) or baseline (*t*(9) = 8.0, *p* < 0.001, Cohen’s *d* = 1.37). Adaptation to matching stimuli also increased thresholds compared to baseline (*t*(9) = 4.2, *p* < 0.005, Cohen’s *d* = 1.34), which may be due to gain changes in binocular integration neurons (see Discussion). The behavioral effects, when plotted as IOCD sensitivity (Fig. 5b), show the same qualitative pattern of results as neural effects of adaptation (Fig. 3b). This similarity suggests that the neural signals we measured with SSVEP may be important for behavior, specifically for the perceptual suppression of one eye’s image, as arises in binocular rivalry.

## Discussion

### Conflict-sensitive neurons in visual cortex

Four distinct results support the conclusion that human early visual cortex contains neurons that respond more strongly when stimuli contain interocular conflict than when they do not: 1) Such neurons will evoke intermodulation frequencies in the EEG, since they necessarily combine signals from both eyes, and their activity should rise as IOCD increases. This matches the observed rise in EEG intermodulation response as a function of IOCD in our results from the baseline condition. 2) Adaptation to conflict should reduce response in these neurons, abolishing the increase in intermodulation response. This effect of adaptation to conflict was also observed in our EEG data. 3) Effects of adaptation should be different following adaptation to matching stimuli, which would not affect conflict-sensitive neurons. We observed a strong trend for this difference. 4) Finally, effects of adaptation to conflict should be different in the fundamental frequencies, since those responses are likely dominated by monocular neurons and binocular integration neurons (see below). This last prediction was also confirmed in our EEG data.

Said and Heeger^12^ proposed that conflict-detecting neurons play an important role in binocular rivalry. They considered traditional models of rivalry based on mutual suppression of neurons tuned for both eye and orientation^8–11,21^ and found that they over-predict the amount and strength of perceptual rivalry when non-conflicting patterns are presented to the two eyes. Consider, for example, the case when a horizontal grating is presented to the left eye, while a vertical one is presented to the right eye. This generally results in perceptual rivalry, and traditional models predict that it arises from mutual “winner-take-all” suppression between neurons that prefer horizontal orientations in the left eye and neurons that prefer vertical ones in the right eye. However, these models make an incorrect prediction for a second case, when identical plaid patterns are presented to each eye. Here, the same winner-take-all suppression would take place between neurons that prefer horizontal in the left eye and neurons that prefer vertical in the right eye (along with additional suppression between other pairs of neurons), predicting strong perceptual suppression. While this stimulus does indeed produce some perceptual suppression, termed monocular rivalry^22^, it is not nearly as strong as the winner-take-all models predict.

The addition of conflict-detecting neurons allowed Said and Heeger’s model to correctly predict only small amounts of interocular suppression for binocular plaids^12^ (see also^4^). They provided some empirical support for their model with behavioral results^12^. Our psychophysical results provide additional behavioral support for the existence of such conflict detecting neurons.

Prior psychophysical work has also found evidence for similar “ocular-opponency” mechanisms^23^ including studies that showed such neurons can affect perception^24–26^. Most of this past work, however, focused on binocular combination, and the role of the opponency mechanisms in interocular suppression remained largely unclear.

Our SSVEP results provide the strongest neurophysiological support to date for models of suppression that include conflict-detecting neurons. A handful of past physiological studies in nonhuman primates found evidence for neurons that received opposing input from the two eyes^27–30^, although this past work did not propose a clear functional role for them, and did not measure human cortex.

An alternative account of our data is that the increase in intermodulation frequencies with increasing conflict was due to a rise in mixed percepts, for example, piecemeal rivalry, where, subjects perceive adjacent patches dominated by different eyes. For mixed percepts, neurons that integrate information from adjacent patches could give rise to intermodulation energy. This is unlikely to explain our main pattern of results, however. Past work from two labs has clearly shown that adapting to rivalry actually increases mixed percepts^12,31^, and so intermodulation response from mixed percepts should rise following adaptation to conflict. Instead we found a decrease in intermodulation response, as would be expected if mechanisms sensitive to conflict were reducing their responsiveness.

### Binocular integration in human cortex

Several aspects of our data suggest that cortex also contains binocular integration neurons, that combine non-conflicting information from the two eyes. Such neurons are present in most models of binocular rivalry^6,8–10^. They often represent the output of the model, as it is widely believed that human visual perception does not have access to monocular signals.

Binocular integration neurons are the likely source of the measured intermodulation response to stimuli with no conflict (IOCD = 0), which should silence conflict-sensitive neurons; though some intermodulation response from conflict-sensitive neurons might be still be elicited due to moment-by-moment changes in eye-specific adaptation and neural noise. Since integration neurons combine information from the two eyes, they would be expected to generate robust intermodulation responses.

Additionally, integration neurons may account for our finding that adaptation to matching stimuli reduced overall levels of intermodulation response, compared to the no adaptation baseline and adaptation to conflict. Such a reduction would be expected if adapting to matching stimuli reduced the responsiveness of binocular integration neurons. These neurons would likely be active in both IOCD = 0 and IOCD = 0.2 conditions, since the latter retains significant matching information from the two eyes. For example, even for IOCD = 0.2 the left and right eyes both view gratings tilted at 45 degrees; the conflict arises from the fact that the contrast of that grating is 20% lower in one eye than in the other.

Integration neurons may also explain why, in our behavioral experiment, adaptation to matching stimuli increased suppression thresholds compared to our no adaptation baseline. The behavioral task asked subjects to judge whether one orientation appeared suppressed compared to the other. This judgment likely made use of signals from binocular integration neurons, which compute the total strength of input for a given orientation. Reduced activity following adaptation of integration neurons would be expected to increase the difficulty of this task, which in turn would raise the amount of conflict required for subjects to report perceptual suppression.

Finally, we also observed that the amplitudes of response at the fundamental frequencies decreased as IOCD increased. The decreased SSVEP signal could arise for at least two different reasons: as IOCD increases, 1) one component grating was lowered in contrast in each eye, and so total stimulus contrast decreased, which could lower responses in many types of neurons, and 2) there was greater interocular suppression, which would lead to lower response from binocular integration and monocular neurons and in turn their contribution to fundamental frequencies.

### Caveats

Importantly, evidence for conflict-sensitive neurons does not rule out the possibility of winner-take-all interocular competition. In fact, the Said and Heeger model simply supplements such competition, implemented via a normalization computation, with gating from conflict-sensitive neurons. Likewise, our results do not exclude winner-take-all competition; it is rather the case that our overall pattern of results cannot be explained by traditional models without additional mechanisms. Our interpretation suggests a role for conflict-sensitive neurons, but it is in principle possible that other additions to winner-take-all models could account for the data.

The IOCDs used in the test stimuli in our EEG experiment were below levels at which observers experience perceptual suppression. We made this choice because at higher levels of IOCD, activity in conflict-sensitive neurons appears to asymptote, making their responses difficult to disentangle from binocular integration neurons which also produce intermodulation energy (see^13^). The adapting stimuli in both experiments, on the other hand, used maximal IOCD (1.0). This maximized neural adaptation and the chances of seeing it’s effects in our data. Importantly, our behavioral data show that the same adaptation condition that reduced the SSVEP marker of conflict-sensitive neurons also raised perceptual suppression thresholds at higher IOCD values.

Nevertheless, the amount of conflict used in the EEG experiment (IOCD = 0.2) was well below the threshold levels measured in the behavioral experiment (IOCD = ~0.6). It is possible that different neural mechanisms might be responsible for processing lower and higher IOCDs, however the single mechanism proposed here provides a parsimonious account of all our results.

### Conflict Resolution in Visual Cortex

The conflict-sensitive neurons measured here are likely located in early visual cortex (i.e. areas V1–V4) since that is the general origin of visual SSVEPs in response to simple stimuli^17,20^, and the topography of our signals is consistent with occipital sources. Future work could use either higher-resolution, cortically referenced SSVEP^32,33^ or functional MRI to determine specifically which early visual areas contain conflict-sensitive neurons.

Li and Atick^34^ first proposed a computational model of how conflict-detecting neurons could be used in conjunction with binocular summation neurons for efficient binocular processing in general. Subsequent studies have demonstrated that incorporating conflict-detecting neurons into models of binocular computation can not only explain binocular rivalry^12^ but also perception of orientation^25^, motion direction^24^, and color^26^, all of which conformed with the predictions made by conflict-sensitive channels. A recent study also found evidence that neurons similar to conflict-detectors could serve to suppress possible interpretations of disparity (“what-not” neurons)^35^. Testing the shared bases for neural mechanisms of suppression will require further development of empirical, theoretical, and modeling approaches that integrate binocular rivalry and stereo vision.

In sum, our results strongly support the hypothesis that conflict-sensitive neurons play a key role in interocular perceptual suppression. It is possible that many types of perceptually ambiguous stimuli are resolved using mechanisms similar to interocular conflict^36–38^. For example, the “Necker cube” may produce competing representations in cortex, corresponding to each of its two interpretations. Resolving this, and other perceptual competitions, may also make use of neurons that explicitly signal the presence of neural conflict.

## Methods

### Subjects

Seventeen subjects (ten females, age: 24 ± 6 (SD) years) participated in Experiment 1. Four of the subjects from Experiment 1, and seven new subjects (three females, age: 24 ± 4 (SD) years) participated in Experiment 2. Fourteen subjects from Experiment 1 and four subjects from Experiment 2 had prior experience with binocular rivalry from other studies. Regardless of their experience with rivalry, all subjects in both experiments were demonstrated basic binocular rivalry for 10 minutes prior to participating in the adaptation experiment. This involved dichoptic presentation of orthogonally oriented gratings and subjects reporting their perception using one of three buttons: one for each grating, and one for a mixture of the two.

For Experiment 1, we removed one subject from analysis because their performance on the IOCD discrimination task (see below) was below chance, bringing our sample size to *N* = 16. For Experiment 2, one subject was also removed from analysis, as they did not exhibit rivalry alternations during the aforementioned rivalry training task, bringing our sample size to *N* = 10. All subjects had normal or corrected to normal visual acuity.

Subjects provided written informed consent to participate in the study in accordance with a protocol approved by the University of Minnesota IRB. All experimental procedures were performed in accordance with this protocol.

### Display

Stimuli were generated using Psychophysics toolbox (PTB–3; RRID:SCR_002881) [45–47] in MATLAB (RRID:SCR_001622; The MathWorks Inc., MA). For Experiment 1 stimuli were presented on an ASUS VG248 monitor (refresh rate 144 Hz), and Experiment 2 on an HP LP2065 (refresh rate 75 Hz). The monitors’ luminance gamma curves were measured using a Photoresearch PR-655 and the luminance was linearized in software to ensure correct display of stimulus intensity. Subjects viewed all stimuli through a custom-built mirror stereoscope.

### Stimuli

In Experiment 1, subjects viewed gratings that had a spatial frequency of 1 cycle/degree, and were presented in circular patches of 6 degrees visual angle diameter, centered at fixation. In Experiment 2, the component gratings had spatial frequency of 1.1 cycle/degree and 5 degrees diameter.

To measure effects of interocular conflict, we used a stimulus manipulation called interocular contrast difference (IOCD) that parametrically introduces conflict, inducing percepts ranging along a spectrum from fusion to strong suppression (Fig. 1a)^13^. Stimuli were comprised of square-wave gratings presented to the two eyes. Each eye viewed the sum of two orthogonal gratings, oriented at + 45 degrees and –45 degrees. At one extreme both gratings were presented at 0.5 contrast to each eye, forming a fused plaid. To introduce conflict, the contrast values of one grating in the left eye and the orthogonal grating in the right eye were reduced, while that of the other grating in each eye remained at 0.5. The orientation of the higher and lower contrast gratings were randomized across trials. The reduced contrast gratings had contrast of 0.5*(1 – *f*_*c*_), where the factor *f*_*c*_ parameterizes different levels of IOCD. Thus, for the IOCD at *f*_*c*_ = 0 (from here on referred to as IOCD = 0), the two eyes viewed identical plaids (Fig. 1a, top), and for IOCD = 1, each eye viewed only one of the two orthogonal gratings. For intermediate IOCDs a different grating in each eye had reduced contrast, producing varying degrees of interocular conflict (Fig. 1a, bottom). As IOCD increased, perception gradually changed, starting from clear fused plaids at IOCD = 0, to clear suppression, in the form of rivalry, for high IOCDs^13^. In Experiment 1, we measured SSVEP responses to test stimuli at two different IOCD levels—0.0 and 0.2. Although clear perceptual suppression cannot usually be discerned until a higher IOCD (~0.5–0.7), our previous findings showed that even the slight increase in IOCD from 0.0 to 0.2 is sufficient to induce a neural signature of binocular conflct. In Experiment 2, the IOCD was varied to determine a threshold for interocular suppression (see below).

In both experiments, subjects viewed stimuli in one of three adaptation conditions: no adaptation (or baseline), adaptation to conflicting stimuli (rivaling gratings), and adaptation to matching stimuli (fused gratings). The general paradigm used in the adaptation experiment is shown in Fig. 1b. The two dots in each panel represent the fixation points for the left and right eyes respectively. The three columns show the three adaptation conditions. For baseline there was no adaptor and subjects saw a mean gray field between test trials. For conflicting adaptation, one grating was presented to the left eye, and the orthogonal grating was presented to the right eye; this is the state of maximum conflict with IOCD = 1 and was used to maximize adaptation. For matching adaptation, identical gratings were presented to the two eyes.

### Procedures

#### Experiment 1: SSVEP Adaptation

Previous results found an increase in certain SSVEP components as stimulus conflict was increased (IOCD 0 to 0.2), which suggested the presence of conflict-sensitive neurons^13^. We used these same two IOCD values as test stimuli in Experiment 1.

In order to ensure subjects were maintaining attention on the stimulus, we asked them to perform a task discriminating between the stimuli with their two IOCD values – 0 and 0.2. The stimulus with IOCD of 0.2 has lower total contrast than the stimulus with IOCD of 0, and for the 2 s presentation duration used here all subjects perceived it as generally appearing lower in contrast than IOCD 0. Subjects responded by pressing one of two buttons to indicate whether they saw a “higher” (IOCD = 0) or a “lower” contrast plaid (IOCD = 0.2). For these low IOCD values, all stimuli appear fused, and IOCD of 0.2 occasionally appears lustrous^2,39^.

To ensure subjects could perform this task, before the main experiment we trained them to discriminate small changes in IOCD from 0. This training consisted of 2–4 runs of 50 trials, where each trial was either a fused plaid (IOCD = 0) or a stimulus with higher IOCD, controlled by a staircase procedure. Subjects again judged the overall contrast of the pattern and pressed a button to indicate whether they had seen a higher (IOCD = 0) or lower (IOCD > 0) contrast pattern. All subjects could successfully discriminate between IOCD 0 and 0.2 prior to the adaptation experiment. To produce SSVEPs, the test stimuli in all trials alternated with a mean gray field at 7.2 Hz (*f*_1_) in the left eye and 12 Hz (*f*_2_) in the right eye.

Adaptation runs followed the pattern depicted in Fig. 1b, with an initial 60-s adaptation period followed by 20 repetitions of 7 s “top-up” adaptation and a 2 s test. For adaptation to conflict, subjects viewed orthogonal ( ± 45 degrees) gratings in each eye, which alternated between orthogonal orientations every 1 s (Fig. 1b). For adaptation to matching stimuli, they viewed identical gratings in both eyes that again alternated between orthogonal orientations every 1 s.

After initial adaptation, there were 20 trials where a test stimulus was presented, separated by an additional 7 s of “top-up” adaptation. During the baseline runs, there was no initial adaptation or top-up period, and trials were simply presented with a 950–1150 ms mean field presentation between them. In all runs, trials were equally and randomly divided between each level of IOCD (0 and 0.2).

The main experiment comprised 15 runs, with first 3 runs of baseline, followed by 6 runs each of adaptation to conflict and matching stimuli. The adaptation runs were presented in alternation with their order counterbalanced across subjects. The runs were self-paced with subjects starting each run by pressing a button. Between each adaptation to conflict and matching stimuli run there was a mandatory gap of 60 s during which subjects could not initiate the next run. This gap provided a period of recovery from the adaptation produced by the previous run.

#### EEG data acquisition and analysis

In Experiment 1, EEG data were acquired using an ANT (Advanced Neuro Technology, The Netherlands) system with a sampling frequency of 1024 Hz. The topography of the 36 channels locations that we acquired is shown in Fig. 2b.

Analysis was conducted using EEGLAB (RRID:SCR_007292)^40^ and customized MATLAB code13. Raw EEG data were band-pass filtered between 1–59 Hz. Independent Component Analysis (ICA) implemented via EEGLAB, was used to remove ocular and muscle artifact^41^. Additional artifacts were identified automatically (voltage exceeding ± 100 µV within a 200 ms window), and trials containing them were excluded from further analysis.

We analyzed our SSVEP results at three frequencies: the two fundamental stimulus frequencies, *f*_1_ and *f*_2_, and the “beat” intermodulation frequency *f*_2_–*f*_1_^13^. Other harmonic and intermodulation frequencies did not consistently yield high signal levels. The response at these three frequencies was estimated in trials from each of the six possible stimulus conditions (two IOCD levels x three adaptation conditions). Energy at each frequency was calculated using MATLAB’s Fast Fourier Transform (FFT) implementation. To determine the analysis time window, we computed the variance between observers, averaged across conditions, and considered the window with the minimum variance (which was 0.8 s) to be the optimal analysis window. Note however that epoch lengths ranging between 0.6–2 s all showed a similar pattern to the reported results.

Time-series data across trials was first averaged over all measured occipital and parieto-occipital electrodes (O_1_, O_2_, O_z_, PO_3_, PO_3_, PO_4_, PO_5_, PO_6_, PO_7_, PO_8_) in the time domain before we performed the frequency domain analyses, computing the FFT. Because our different stimulus frequencies did not evenly divide into our analysis window, their peaks fell between integer values of the FFT we computed. Accordingly, we upsampled the FFT to higher resolution. However, noise in the data produced peaks in the FFT at slightly different bins for different subjects. To overcome this issue we took the frequency bin with the maximum amplitude within a ± 0.14 Hz range around our tagged frequency, and used that amplitude as our estimate of signal strength. We then calculated a signal-to-noise ratio (SNR) metric from the FFT as the ratio of that value to the mean amplitude of all frequencies between ± 1.32 and ± 1.40 Hz around the target frequency (as noise floor). The nearest noise frequency of 1.32 Hz was chosen to be outside the resolution of our integer FFT frequencies plus the window within which we took the maximum response (1/0.8 + 0.07 = 1.32 Hz). For all sessions, the lowest SNR at the SSVEP frequencies was observed at the centro-parietal electrodes, and so, we used these as reference electrodes. Data were also analyzed using an average-of-all-electrodes reference and showed overall the same pattern of results but with larger variability.

#### Experiment 2: Behavioral Adaptation to Conflicting and Matching gratings

This experiment measured perception as a function of IOCD, in order to obtain thresholds where subjects’ perceived suppression. This transition is not abrupt; rather the amount of suppression perceived increases gradually as a function of IOCD^13^. We asked subjects to judge the contrast of the two component gratings of the plaid, determining whether the two components appeared equal in contrast or not. Because the decreases in contrast that comprise the IOCD are symmetric across the two eyes, the components of the plaid appeared to have equal total contrast when the stimulus was perceived as fused. On the other hand, if either of the two eyes was suppressed, there should be a difference in perceived contrast between the two components. Thus, responses in the contrast judgment reflected whether one eye was suppressed.

Subjects had a 2.5 s window following the presentation of a test stimulus to indicate by pressing one of two buttons whether they perceived the components of the test plaid as having equal contrast, which we interpreted as fusion, or as differing in contrast, interpreted as suppression. To aid performance, subjects were initially given brief training in discriminating between plaids whose components had either identical (IOCD = 0) or an identical contrast reduction in both eyes, using a two-alternative-forced-choice task.

For the main experiment, subjects performed the contrast judgment task with stimuli that varied in IOCD. We defined the point at which subjects reported seeing fusion and suppression equally often as the suppression threshold. IOCD was initially set to 0.5, and was updated after each trial based on subjects’ response using a one-up-one-down staircase procedure: If subjects reported the IOCD stimulus components as having equal contrast, the IOCD value was incremented by 0.02 and if they reported unequal contrast the IOCD value was decremented by the same amount.

There were again three sets of runs: baseline, adaptation to conflicting (rivaling) stimuli, and adaptation to matching (fused) stimuli (Fig. 1b). During each run, there were twenty-two trials containing a 750-ms test stimulus presentation. For the baseline runs, trials were separated by a 2.5 s interval of mean field presentation. During adaptation runs, there was an initial adaptation period of 60 s and each trial began with an 8 s top-up adaptation period.

Subjects completed 18 runs of trials: Four runs of the baseline condition followed by 6 runs each of adaptation to fusion and conflict (randomly interleaved), followed by another 2 runs of the baseline condition. Runs were self-paced with subjects initiating them by pressing a button. Between the adaptation runs there was a mandatory gap of 60 s, which provided a recovery period from the previous adaptation condition.
